# Cu–CeO_2_ NP-loaded HAMA hydrogel patches for promoting high-quality cutaneous wound healing

**DOI:** 10.3389/fbioe.2026.1872283

**Published:** 2026-08-05

**Authors:** Jiahao Ji, Jiamin Ni, Fei Chang, Like Qian, Hongyi Zhang, Dongli Chen

**Affiliations:** 1 The Affiliated Zhangjiagang Hospital of Soochow University, Suzhou, China; 2 Department of Plastic Surgery, Ear Reconstruction Institute, Shanghai East Hospital, School of Medicine, Tongji University, Shanghai, China

**Keywords:** biomaterials, HAMA hydrogel, nanoparticles, reactive oxygen species, wound healing

## Abstract

**Background:**

Wound healing is a common issue in dermatology and plastic surgery practice and is jointly regulated by multiple factors, including cell proliferation, inflammatory responses, oxidative stress, bacterial infection, and tissue remodeling. Complex wounds still face challenges such as delayed repair, persistent inflammation, increased infection risk, and scar formation, while conventional treatments are difficult to achieve coordinated intervention across multiple stages. Therefore, developing biomaterials with pro-repair, anti-inflammatory, antibacterial, and healing quality-improving functions is of great significance.

**Objective:**

This study aimed to construct a Cu–CeO_2_ nanoparticle (NP)-loaded hyaluronic acid methacrylate composite hydrogel patch, named Cu–CeO_2_-loaded HAMA (CCH), to improve the wound repair microenvironment and promote high-quality wound healing through multifunctional synergistic effects.

**Methods:**

Cu–CeO_2_ NPs were synthesized using a hydrothermal method and loaded into HAMA hydrogel to prepare CCH hydrogel patches. *In vitro* experiments were performed to evaluate their biocompatibility, cell proliferation- and migration-promoting abilities, antioxidative/anti-inflammatory properties, nanozyme activity, and antibacterial effects. Meanwhile, a mouse full-thickness skin wound model was established to further verify the *in vivo* wound healing-promoting efficacy of CCH hydrogel patches.

**Results:**

Cu–CeO_2_ NPs and CCH hydrogel patches were successfully prepared. *In vitro* results showed that Cu–CeO_2_ NPs exhibited favorable biocompatibility, promoted fibroblast proliferation and migration, enhanced endothelial cell viability, and facilitated macrophage polarization toward the M2 phenotype. In addition, these NPs exhibited reactive oxygen species (ROS)-scavenging capacity and hydrogen peroxide (H_2_O_2_) decomposition-mediated oxygen generation ability, and markedly inhibited methicillin-resistant *Staphylococcus aureus* (MRSA) and *Escherichia coli* (*E. coli*). *In vivo* results further demonstrated that CCH hydrogel patches accelerated wound closure, improved healing quality, and attenuated scar-like changes in mice.

**Conclusion:**

This study developed Cu–CeO_2_ NP-loaded CCH hydrogel patches that can synergistically regulate the wound microenvironment through multiple effects, including pro-repair, antioxidative/anti-inflammatory, antibacterial, and tissue remodeling-improving activities, providing a promising biomaterial strategy for high-quality repair of complex skin wounds.

## 1 Introduction

The skin is the largest organ of the human body and serves as a critical physiological barrier protecting against mechanical injury, chemical stimuli, and pathogenic microbial invasion (Harris-Tryon and Grice, 2022; Baker et al., 2023). Various factors, including trauma, surgical incisions, burns, and chronic diseases such as diabetes mellitus (DM), can disrupt the structural integrity of the skin and result in different types of wounds (Falanga et al., 2022; Sen, 2023). Wound healing is a continuous and highly complex biological process involving multiple phases, including inflammatory response, cell proliferation and migration, angiogenesis, extracellular matrix (ECM) deposition, and tissue remodeling, which are collectively regulated by fibroblasts, immune cells, endothelial cells, and the local microenvironment (Eming et al., 2021). However, several critical challenges remain in the repair of complex wounds. First, insufficient proliferation and migration of fibroblasts can delay granulation tissue formation, thereby impairing wound healing. Second, local oxidative stress and hypoxic microenvironments may exacerbate cellular damage and hinder tissue regeneration. Third, persistent immune-inflammatory responses, particularly the maintenance of pro-inflammatory macrophage phenotypes, can further impede wound healing. Fourth, bacterial infection may aggravate local inflammation and lead to chronic non-healing wounds (Diller and Tabor, 2022; Huang et al., 2022; Hunt et al., 2024). Therefore, the development of a multifunctional composite material capable of simultaneously promoting cellular repair, alleviating oxidative stress, modulating the inflammatory microenvironment, and inhibiting bacterial growth is of great significance for achieving rapid and high-quality wound healing.

In recent years, nanomaterials have attracted extensive attention in the field of wound repair due to their unique physicochemical properties and tunable biological functions. Studies have shown that Fe_3_O_4_ nanoparticles (NPs) can promote fibroblast proliferation, thereby accelerating wound healing (Wang et al., 2025). However, Fe_3_O_4_ NPs may also induce pro-inflammatory responses, promoting macrophage polarization toward the M1 phenotype, which can exacerbate wound inflammation and, to some extent, delay wound healing or even increase the risk of scar formation. In contrast, cerium dioxide nanoparticles (CeO_2_ NPs) possess reversible Ce^3+^/Ce^4+^ redox cycling capability, enabling them to mimic multiple antioxidant enzyme activities and scavenge excessive reactive oxygen species (ROS) in wounds, thereby alleviating oxidative stress–induced damage and improving the inflammatory microenvironment (Corsi et al., 2023). For instance, Cheng et al. (Cheng et al., 2021)reported that hydrogel dressings loaded with CeO_2_ NPs could promote cutaneous wound healing by scavenging ROS, reducing oxidative stress, and modulating the wound microenvironment. Nevertheless, the application of CeO_2_ NPs alone in complex wounds remains limited. Their antibacterial activity, pro-angiogenic capacity, and ability to regulate multiple stages of wound repair are relatively insufficient, making it difficult to simultaneously meet the multifaceted demands of complex wounds, including antioxidation, anti-infection, tissue regeneration, and immune modulation (Xue et al., 2024). Therefore, further optimization of CeO_2_-based nanosystems and enhancement of their integrated biological functions represent an important direction for improving their potential in wound healing applications.

Copper plays an important role in tissue repair and antibacterial therapy. Appropriate concentrations of Cu^2+^ not only exert antibacterial effects by disrupting bacterial membrane integrity, interfering with bacterial metabolism, and inducing oxidative damage, but also participate in angiogenesis, cell proliferation, and tissue regeneration processes (Huang et al., 2024). Previous studies have demonstrated that hydrogels reinforced with Cu^2+^-coordinated nanoparticles can effectively promote cutaneous wound healing by improving the oxidative stress microenvironment, inhibiting bacterial proliferation, enhancing angiogenesis, and accelerating tissue repair (Jin et al., 2023). However, the Fenton reaction associated with Fe_3_O_4_ and its potential pro-inflammatory effects may increase the local oxidative stress burden, thereby limiting its application in wound healing to some extent (Luo et al., 2024; Nowak-Jary and Machnicka, 2024). Based on these considerations, the combination of Cu with CeO_2_ NPs is expected to form a milder and multifunctional composite nanoplatform. On the one hand, CeO_2_ NPs can enhance ROS scavenging and anti-inflammatory repair through the Ce^3+^/Ce^4+^ redox cycling system; on the other hand, the introduction of Cu can further endow the material with antibacterial and pro-angiogenic properties, thereby synergistically improving the wound microenvironment and promoting tissue regeneration (Jiang et al., 2024; Şener et al., 2024; Xue et al., 2024). Therefore, Cu–CeO_2_ composite NPs may simultaneously exert multiple functions during wound healing, including antioxidative, anti-inflammatory, antibacterial, and pro-regenerative effects.

In addition to the active nanomaterials themselves, an appropriate delivery carrier is also crucial for improving local therapeutic efficacy in wound repair. Hyaluronic acid methacrylate hydrogel (HAMA) exhibits excellent biocompatibility, hydrophilicity, and photo-crosslinking properties, enabling it to provide a moist wound healing environment and serve as a localized delivery platform for nanoparticles or therapeutic agents, thereby achieving stable retention and sustained release of active components at the wound site (Gong et al., 2022; Chang et al., 2023). Moreover, the three-dimensional porous network structure of hydrogels facilitates moisture retention, nutrient exchange, cell migration, and tissue regeneration (Zhang et al., 2025). Therefore, loading Cu–CeO_2_ NPs into HAMA hydrogels is expected to construct a composite dressing system that integrates structural support with biological activity, thereby enhancing its therapeutic efficacy in complex wound repair. In this study, HAMA was selected as the delivery matrix for Cu–CeO_2_ nanoparticles mainly because of its advantages in wound repair. HAMA has good biocompatibility, hydrophilicity, and low immunogenicity, which help maintain a moist wound environment and support cell migration, nutrient exchange, and tissue regeneration (Altunbek et al., 2024). After methacrylation, HAMA can be rapidly photocrosslinked into a stable three-dimensional porous network. This not only makes it convenient to prepare hydrogel patches with a controlled shape, but also helps achieve uniform loading and local retention of Cu–CeO_2_ nanoparticles. Compared with commonly used hydrogels such as GelMA, GelMA provides better cell-adhesive properties, but it is derived from gelatin/collagen and may degrade relatively quickly in the enzyme-rich wound environment, with possible batch-to-batch variation (Yue et al., 2015). In contrast, HAMA offers better moisture retention, lower immunogenicity, and more controllable photocrosslinking, which better fits the design of this study for wound coverage, local hydration, and sustained nanoparticle delivery. In addition, our results showed that the CCH hydrogel maintained good mechanical properties and structural stability after loading with Cu–CeO_2_ nanoparticles, indicating that HAMA can serve as an effective nanoparticle delivery platform and provide a material basis for sustained antioxidant, anti-inflammatory, antibacterial, and pro-repair effects at the wound site.

Based on the aforementioned rationale, in this study we designed and constructed a Cu–CeO_2_ nanoparticle-loaded HAMA composite hydrogel patch (CCH). Specifically, Cu–CeO_2_ NPs were first synthesized via a hydrothermal method and systematically characterized in terms of morphology, elemental composition, particle size distribution, and dispersion stability using transmission electron microscopy (TEM), high-angle annular dark-field imaging (HAADF), selected area electron diffraction (SAED), energy-dispersive spectroscopy (EDS) elemental mapping, dynamic light scattering (DLS), and zeta potential analysis. Subsequently, Cu–CeO_2_ NPs were uniformly incorporated into HAMA hydrogel to fabricate the CCH hydrogel patch, and its porous network structure and elemental distribution were verified by scanning electron microscopy (SEM) and EDS mapping. Furthermore, *in vitro* experiments were conducted to systematically evaluate its biosafety, cell proliferation and migration–promoting effects, reactive oxygen species (ROS) scavenging capacity, H_2_O_2_ decomposition–mediated oxygen generation capability, regulation of macrophage polarization, and antibacterial activity. Finally, a full-thickness skin wound model in mice was established to assess the *in vivo* efficacy of the CCH hydrogel patch in promoting wound healing and improving healing quality. It is anticipated that the CCH hydrogel patch can exert synergistic effects through “pro-repair, antioxidative/anti-inflammatory, antibacterial, and healing quality improvement,” thereby providing a novel strategy and experimental basis for high-quality repair of complex cutaneous wounds.

## 2 Methods

### 2.1 Synthesis of Cu–CeO_2_ nanoparticles (NPs)

The preparation of Cu–CeO_2_ composite NPs mainly involved several steps, including SiO_2_ template construction, hollow CeO_2_ (HCeO_2_) formation, surface positive charge modification, copper nanoparticle synthesis, and composite assembly. First, SiO_2_ nanospheres were synthesized using the Stöber method. Briefly, ethanol, deionized water, and ammonia solution were mixed at a defined ratio, followed by the addition of tetraethyl orthosilicate (TEOS) under continuous stirring, and the reaction was maintained for 4–8 h. The resulting precipitate was collected by centrifugation and repeatedly washed with ethanol and deionized water to obtain the SiO_2_ templates. Subsequently, 35 mg of SiO_2_ was dispersed in deionized water, followed by ultrasonication and mechanical stirring. Then, 150 mg of cerium acetylacetonate and 171 mg of urea were added, and the mixture was further stirred to ensure homogeneous mixing. The reaction system was then transferred into a Teflon-lined autoclave and subjected to hydrothermal treatment at 160 °C for 6–8 h to obtain HCeO_2_ NPs. To enhance subsequent composite efficiency, 40 mg of HCeO_2_ was dispersed in 20–30 mL of deionized water, followed by the addition of 200 mg of poly (allylamine hydrochloride) (PAH) for surface modification. After stirring for 3 h, the product was collected by centrifugation to obtain positively charged HCeO_2_ NPs. In parallel, 10 mmol of copper chloride was dissolved or dispersed in 50 mL of deionized water and preheated at 80 °C under stirring for 15 min. Subsequently, 50 mL of 400 mM L-ascorbic acid solution was slowly added dropwise, and the reaction was continued at 80 °C for 16–18 h to generate copper NPs. After completion, larger aggregates were removed by centrifugation at 6,577 *g* for 20 min, and the supernatant was collected and dialyzed in a 10 kDa dialysis bag for 48 h to remove unreacted small-molecule impurities. Finally, 40 mg of PAH-modified HCeO_2_ was added to the copper NP dispersion and continuously stirred for 12–14 h to ensure sufficient interaction and assembly. The resulting product was collected by centrifugation and washed three times with deionized water to obtain the final Cu–CeO_2_ composite NPs.

### 2.2 Characterization of Cu–CeO_2_ NPs and CCH hydrogel patches

Transmission electron microscopy (TEM) was used to observe the morphology and dispersion state of Cu–CeO_2_ NPs, and high-angle annular dark-field scanning transmission electron microscopy (HAADF-STEM) was further performed to analyze their structural features. Selected area electron diffraction (SAED) was used to evaluate the crystalline structure of the NPs. Energy-dispersive X-ray spectroscopy (EDS) elemental mapping was performed to detect the spatial distribution of Ce, O, and Cu within the NPs, thereby confirming the successful construction of Cu–CeO_2_ composite NPs. The hydrodynamic diameter and surface potential of the NPs were measured using a nanoparticle size and zeta potential analyzer to assess their particle size distribution and colloidal stability.

To evaluate the microstructure of the CCH hydrogel patches, HAMA hydrogel and Cu–CeO_2_ NP-loaded CCH hydrogel samples were lyophilized and then examined by scanning electron microscopy (SEM) to observe their internal porous network structures (Supplementary Figure 1). Pore size was quantitatively analyzed using ImageJ software. In addition, EDS elemental mapping was performed to analyze the distribution of Ce, Cu, and O within the CCH hydrogel, thereby evaluating the loading and dispersion status of Cu–CeO_2_ NPs in the HAMA hydrogel matrix.

### 2.3 Preparation of Cu–CeO_2_ NP-loaded hydrogel patches

A 10% (w/v) HAMA hydrogel precursor solution was prepared and fully dissolved at 37 °C until a homogeneous solution was obtained. Subsequently, Cu–CeO_2_ NPs were added to the HAMA precursor solution and uniformly dispersed within the hydrogel system by repeated pipetting. The resulting mixed solution was transferred into circular molds with a diameter of approximately 1 cm under light-protected conditions. After gently removing air bubbles, the hydrogel was photo-crosslinked under 405 nm visible light irradiation for approximately 30 s. After gelation, the hydrogel was removed from the mold to obtain the Cu–CeO_2_ NP-loaded HAMA composite hydrogel patch.

### 2.4 Cell culture

Human dermal fibroblasts (HDFs), human umbilical vein endothelial cells (HUVECs), and RAW 264.7 macrophages used in this study were purchased from IM-MOCELL (Xiamen, Fujian, China). HDFs and HUVECs were cultured in their respective complete culture media, while RAW 264.7 cells were maintained in Dulbecco’s modified Eagle’s medium (DMEM) supplemented with 10% fetal bovine serum (FBS) and 1% penicillin–streptomycin. All cells were routinely cultured in a humidified incubator at 37 °C with 5% CO_2_, and the culture medium was replaced or cells were passaged as needed according to cell growth status. Before experiments, cells were seeded in appropriate culture plates. After cell attachment and reaching the desired confluence, cells were treated with Control, CeO_2_, or Cu–CeO_2_ treatment solutions for subsequent assays, including cell proliferation, migration, macrophage polarization, and related functional experiments.

### 2.5 Live/dead cell staining assay

HDFs were seeded in 24-well plates at a density of 5 × 10^4^ cells/well and cultured in a humidified incubator at 37 °C with 5% CO_2_ to allow sufficient cell attachment. Subsequently, the medium was replaced with complete medium containing different concentrations of Cu–CeO_2_ NPs (0, 6.25, 12.5, 25, 50, 100, 200, and 400 μg mL-1), with three replicate wells per group. After incubation, the culture medium was removed, and the cells were gently washed twice with phosphate-buffered saline (PBS) to eliminate residual medium and unbound NPs. Then, live/dead staining working solution containing 2 μM Calcein-AM and 4 μM propidium iodide (PI) was added, followed by incubation at 37 °C for 20 min in the dark. After staining, the cells were gently washed again with PBS and observed under a fluorescence microscope. Live cells exhibited green fluorescence after Calcein-AM staining, whereas dead cells were labeled with red fluorescence by PI. The cytotoxicity and *in vitro* biosafety of Cu–CeO_2_ NPs toward HDFs were evaluated by comparing the green fluorescence intensity and red fluorescence signals among groups.

### 2.6 CCK-8 assay for *in vitro* cytotoxicity

The cytotoxicity of Cu–CeO_2_ NPs toward HDFs was further evaluated using the Cell Counting Kit-8 (CCK-8) assay. Briefly, HDFs were seeded into 96-well plates. After cell attachment, complete medium containing different concentrations of Cu–CeO_2_ NPs was added for treatment. After incubation for the indicated time, the culture medium was removed, and the cells were gently washed with PBS. Subsequently, fresh culture medium containing CCK-8 reagent was added to each well, followed by incubation at 37 °C in the dark. After incubation, the absorbance at 450 nm was measured using a microplate reader. The relative cell viability was calculated based on the optical density (OD) values of each group to evaluate the *in vitro* biosafety of Cu–CeO_2_ NPs toward HDFs at different concentrations.

### 2.7 Assessment of cell proliferation and migration

The proliferative capacity of HDFs and HUVECs after treatment with different materials was evaluated using the CCK-8 assay. HDFs or HUVECs were seeded into 96-well plates at an appropriate density. After 24 h of culture to allow cell attachment, Control, CeO_2_, or Cu–CeO_2_ treatment solutions were added, and the cells were further cultured. CCK-8 assays were performed on days 1, 4, and 7. Briefly, 10 μL of CCK-8 solution was added to each well, followed by incubation at 37 °C for 2 h in the dark. The absorbance at 450 nm was then measured using a microplate reader, and the OD values were used to reflect cell viability and proliferation trends.

A wound scratch assay was performed to evaluate the effects of different materials on HDF migration. HDFs were seeded into 24-well plates and cultured until reaching approximately 80%–90% confluence. A sterile 200 μL pipette tip was then used to vertically scratch the cell monolayer to generate a linear wound area with a relatively uniform width, followed by gentle washing with PBS 2–3 times to remove detached cells. Subsequently, Control, CeO_2_, or Cu–CeO_2_ treatment solutions were added, and the cells were further cultured. Changes in the scratched area were observed and photographed under an optical microscope at 0 and 24 h. Finally, the scratch area in each group was quantitatively analyzed using ImageJ software, and the migrated area or migration rate was calculated to assess the effect of Cu–CeO_2_ NPs on HDF migration.

### 2.8 Evaluation of ROS-scavenging capacity and H_2_O_2_ decomposition-mediated oxygen generation

To evaluate the antioxidant properties of Cu–CeO_2_ NPs, their scavenging capacity against multiple reactive oxygen species (ROS) was examined. Different concentrations of CeO_2_ or Cu–CeO_2_ NPs were added to the corresponding ROS detection systems. The scavenging efficiencies against O_2_•^-^, H_2_O_2_, and •OH were determined according to the instructions of the assay kits or experimental protocols, and the scavenging rate of each group was calculated. To further assess the H_2_O_2_ decomposition-mediated oxygen generation capacity of the NPs, CeO_2_ or Cu–CeO_2_ NPs were added to an H_2_O_2_-containing reaction system, and changes in dissolved oxygen concentration were measured at different time points to evaluate their ability to catalyze H_2_O_2_ decomposition and oxygen production.

### 2.9 Flow cytometric analysis of macrophages

RAW 264.7 macrophages were seeded into cell culture plates. After cell attachment, the cells were treated with Control, CeO_2_, or Cu–CeO_2_ treatment solutions. After treatment, cells from each group were collected and washed with PBS. Subsequently, the cells were resuspended in flow cytometry staining buffer and incubated with relevant antibodies, including F4/80 and CD206, in the dark. After staining, the cells were collected by centrifugation and washed with PBS to remove unbound antibodies. Finally, the fluorescence signal distribution of M2 polarization-related markers in macrophages from each group was detected using a flow cytometer and analyzed with FlowJo software, thereby evaluating the regulatory effect of Cu–CeO_2_ NPs on the M2-like reparative phenotype of macrophages.

### 2.10 *In vitro* antibacterial activity evaluation

Methicillin-resistant *Staphylococcus aureus* (MRSA) and *Escherichia coli* (*E. coli*) were selected as representative Gram-positive and Gram-negative bacterial models, respectively. After co-incubation of bacterial suspensions with materials from different treatment groups for a specified period, the bacterial suspensions were serially diluted and evenly spread onto agar plates, followed by incubation at 37 °C to observe colony formation. The inhibitory effects of different treatments on bacterial growth were evaluated based on colony counts or relative bacterial viability. To further observe changes in bacterial morphology and structure after different material treatments, bacterial samples from each group were collected, fixed, gradient-dehydrated, and dried, followed by SEM observation of bacterial micromorphology. The *in vitro* antibacterial performance of CeO_2_ and Cu–CeO_2_ NPs was comprehensively evaluated by integrating agar plate colony formation results, relative bacterial viability, and SEM-based morphological observations.

### 2.11 Establishment and treatment of a full-thickness skin wound model in mice

Female BALB/c mice aged 6–8 weeks were purchased from a laboratory animal supplier. All animal experimental procedures were approved by the Animal Ethics Committee of Biotech-YueKang Life Science (Shanghai) Co., Ltd (YK20260301). Before the experiments, mice were acclimatized and maintained under standard housing conditions throughout the study. For model establishment, mice were anesthetized using inhalational isoflurane anesthesia (3%–4% for induction and 1.5%–2% for maintenance in oxygen). After stable anesthesia was achieved, the dorsal hair was removed, and the surgical area was routinely disinfected. Subsequently, a circular full-thickness skin defect with a diameter of approximately 1 cm was created on the dorsum of each mouse to establish the murine skin wound model. After model establishment, the mice were randomly divided into the Control, CeO_2_, and Cu–CeO_2_ groups and treated with the corresponding hydrogel patches or no hydrogel patch. After the hydrogel patch was placed on the wound, medical adhesive tape was used to fix the patch in position and prevent displacement or detachment after the mice recovered from anesthesia and resumed normal activity. This fixation helped maintain close contact between the hydrogel patch and the wound surface, allowing the loaded Cu–CeO_2_ nanoparticles to be released locally at the wound site. On postoperative day 3, the adhesive tape and residual hydrogel were removed during wound imaging. At this time point, the hydrogel had been largely absorbed or degraded. During the experiment, mice were housed separately to prevent mutual scratching or biting that could affect wound healing. Wounds were photographed on postoperative days 0, 3, 7, and 14. Before imaging, hair around the wound area was removed to allow clearer observation of wound healing and closure. At the end of the experiment, mice were deeply anesthetized with isoflurane (4%–5%) and euthanized by overdose inhalation of isoflurane. Death was confirmed by the absence of respiration and heartbeat before tissue harvesting. Wound tissues together with surrounding skin tissues were subsequently collected for further analyses as required (He et al., 2025).

### 2.12 Mechanical and swelling characterization of hydrogels

The mechanical properties of the hydrogels were evaluated by compression testing. Briefly, HAMA and CCH hydrogels were prepared in cylindrical molds and subjected to unconfined compression using a mechanical testing system. The compressive stress–strain curves were recorded, and the Young’s modulus was calculated from the initial linear region of the curve.

### 2.13 Evaluation of hydrogel swelling behavior

The swelling behavior of the hydrogels was measured by immersing pre-weighed dried hydrogel samples in PBS at 37 °C. At predetermined time points, the samples were removed, gently blotted with filter paper to remove surface water, and weighed immediately. The swelling ratio was calculated as follows: swelling ratio (%) = (Wt − W0)/W0 × 100%, where W0 is the initial dry weight and Wt is the weight of the swollen hydrogel at each time point.

### 2.14 Histological staining analysis

To evaluate the tissue structure after wound repair, wound tissues together with surrounding skin were collected on postoperative day 14 and fixed in 4% paraformaldehyde. The samples were then routinely dehydrated, embedded in paraffin, and sectioned. The tissue sections were stained with hematoxylin and eosin (H&E) and Masson’s trichrome staining. H&E staining was used to observe epidermal regeneration, inflammatory cell infiltration, and overall tissue structure, while Masson staining was used to assess collagen deposition and collagen fiber organization. The stained sections were observed and photographed under a light microscope.

### 2.15 Collagen deposition analysis

To further evaluate collagen deposition in the wound tissue, image analysis was performed on Masson-stained images. Briefly, under the same image acquisition conditions, the regenerated wound areas from each group were selected for analysis, and the Masson-positive collagen areas were evaluated using ImageJ software. The results of the Control group were normalized to 1, and the results of the other groups were expressed as relative values.

### 2.16 Collagen fiber organization analysis

To further evaluate collagen fiber remodeling in the wound tissue, collagen fiber organization was semi-quantitatively assessed based on the regularity and structural organization of collagen fibers in Masson-stained images. All analyses were performed using the same criteria to ensure comparability among groups.

### 2.17 Statistical analysis

Statistical analysis and graphing were performed using GraphPad Prism software, and image quantification was conducted using ImageJ software. All quantitative data were obtained from at least three independent experiments, with no fewer than three parallel samples in each group, and are presented as the mean ± standard deviation (mean ± SD). Comparisons between two groups were performed using an unpaired two-tailed Student’s t-test. Comparisons among multiple groups were conducted using one-way analysis of variance (one-way ANOVA), followed by Tukey’s multiple comparison test. Statistical significance was defined as *p* < 0.05, *p* < 0.01, and *p* < 0.001; ns indicates no statistically significant difference.

## 3 Results and discussion

### 3.1 Synthesis and characterization of Cu–CeO_2_ NPs

**FIGURE 1:**
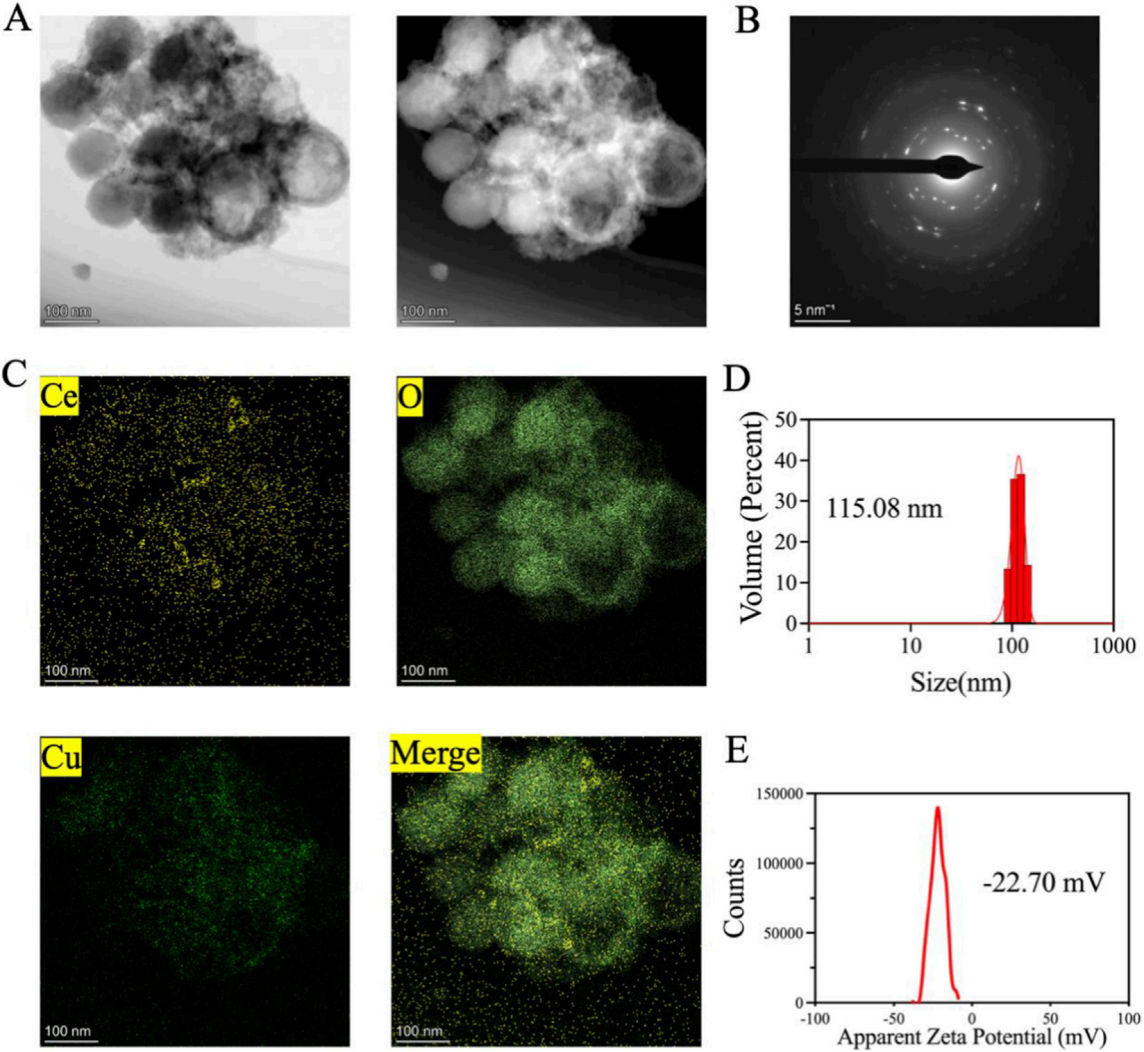
Characterization of Cu–CeO_2_ nanoparticles (NPs). **(A)** Transmission electron microscopy (TEM) image (left, bright-field mode) and high-angle annular dark-field (HAADF) TEM image (right); scale bar = 100 nm. **(B)** Selected area electron diffraction (SAED) pattern of Cu–CeO2 NPs. **(C)** Energy-dispersive X-ray spectroscopy (EDS) mapping scan of Ce (yellow), O (cyan), Cu (green), and merged image; scale bar = 100 nm. **(D)** Particle size distribution of the NPs. **(E)** Zeta potential of the NPs.

To verify the successful preparation and physicochemical properties of Cu–CeO_2_ NPs, their morphology, structure, and dispersibility were systematically characterized. As shown in Figure 1A, TEM images revealed that Cu–CeO_2_ NPs exhibited an overall quasi-spherical morphology with particle sizes in the nanoscale range. Although slight aggregation was observed among particles, the overall dispersibility remained favorable. HAADF images further confirmed the structural uniformity of the NPs. The SAED pattern (Figure 1B) displayed distinct multi-ring diffraction features, indicating that the prepared Cu–CeO_2_ NPs possessed a typical polycrystalline structure consistent with the crystalline characteristics of CeO_2_. EDS elemental mapping (Figure 1C) showed that Ce, O, and Cu were uniformly distributed within the NPs, and the merged image further confirmed the successful incorporation of Cu into the CeO_2_ structure, forming a stable composite nanosystem. DLS analysis (Figure 1D) showed that the hydrodynamic diameter of Cu–CeO_2_ NPs was approximately 115.08 nm with a relatively narrow distribution, suggesting good dispersibility in solution. In addition, zeta potential analysis (Figure 1E) revealed a surface potential of approximately −22.70 mV, indicating favorable colloidal stability of the NP system. Collectively, Cu–CeO_2_ NPs with uniform morphology, good dispersibility, and stable structure were successfully synthesized in this study, providing a material basis for the subsequent construction of functional hydrogel systems and their biological applications.

To further assess the material properties of the hydrogels, we examined their mechanical performance and swelling behavior. Compression testing showed that the HAMA hydrogel had a Young’s modulus of 10.67 kPa, while the Young’s modulus of the CCH hydrogel increased to 13.33 kPa after loading with Cu–CeO_2_ nanoparticles (Supplementary Figure 2). This result suggests that the incorporation of nanoparticles improved the mechanical stability of the hydrogel network, allowing the CCH hydrogel to provide better structural support during wound repair and to maintain a stable environment for cell adhesion, growth, and tissue regeneration. We also evaluated the stability of the hydrogels using a swelling assay. The swelling ratios of both hydrogels gradually increased with time and reached a relatively stable level at 40–60 min. The final swelling ratio of the HAMA hydrogel was approximately 120%, whereas that of the CCH hydrogel was only 45%–50% (Supplementary Figure 3) (Zhou et al., 2026). Compared with the HAMA group, the CCH group showed a much lower swelling ratio. This suggests that the addition of Cu–CeO_2_ nanoparticles made the hydrogel network more compact and restricted the excessive expansion of the polymer chains, thereby reducing water uptake. A lower swelling ratio is helpful for maintaining the shape and mechanical stability of the hydrogel and can reduce volume changes caused by excessive water absorption after application. It may also help the material remain at the wound site for a longer period. These results indicate that the CCH hydrogel has both suitable mechanical properties and good structural stability, supporting its use in wound repair.

In terms of practical wound dressing application, these mechanical and swelling characteristics are closely related to the retention and functional stability of the hydrogel on the wound surface. The moderately increased Young’s modulus of the CCH hydrogel may help the patch maintain its structural integrity after application, reducing deformation or displacement caused by animal movement, wound contraction, or external friction. Meanwhile, the lower swelling ratio of the CCH hydrogel may prevent excessive volume expansion after contact with wound fluid, thereby helping maintain close contact between the hydrogel and the wound bed. This property may also reduce the structural loosening caused by excessive absorption of wound exudate and contribute to prolonged local retention of the material. As a result, Cu–CeO_2_ nanoparticles could be released more steadily within the wound microenvironment and continuously exert antioxidant, anti-inflammatory, antibacterial, and pro-repair effects (Liu et al., 2024). Nevertheless, swelling capacity should not be minimized without limitation, because an appropriate degree of water uptake is still necessary for absorbing wound exudate and maintaining a moist healing environment (Gao et al., 2025). Therefore, the relatively lower but still present swelling capacity of the CCH hydrogel suggests a favorable balance between wound hydration, structural stability, and local retention, which may be beneficial for cutaneous wound repair.

### 3.2 Morphological and elemental distribution characterization of CCH hydrogel patches

**FIGURE 2:**
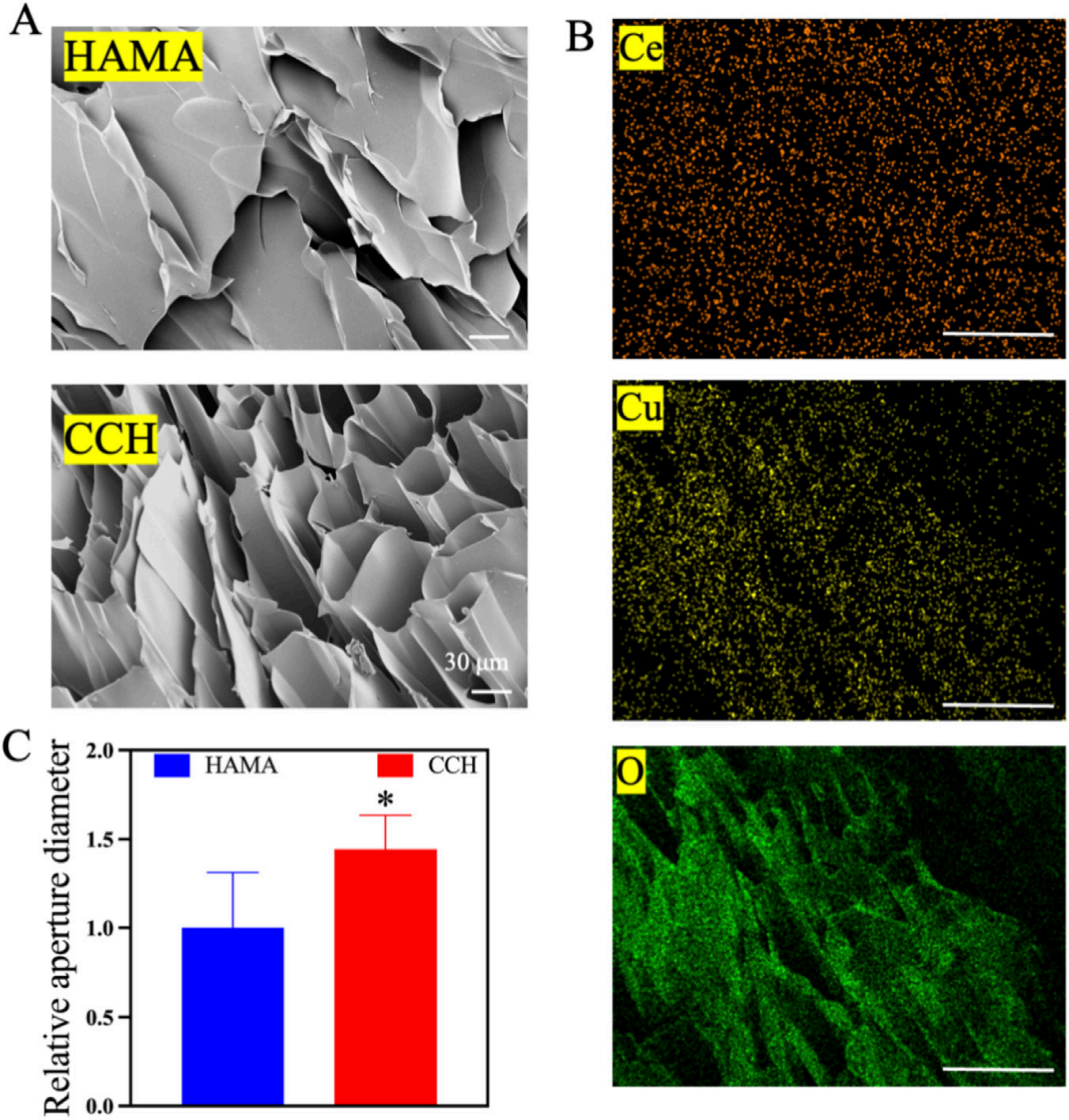
Morphological characterization of Cu–CeO_2_-loaded hyaluronic acid methacrylate (HAMA) hydrogel (CCH) patches. **(A)** Scanning electron microscopy (SEM) images showing the internal porous network structures of pure HAMA hydrogel and CCH hydrogel; scale bar = 30 μm. **(B)** Energy-dispersive X-ray spectroscopy (EDS) elemental mapping showing the distribution of cerium (Ce), copper (Cu), and oxygen (O) within the CCH hydrogel; scale bar = 100 μm. **(C)** Comparison of relative pore size between the HAMA and CCH groups. All data are presented as mean ± standard deviation (SD) (n = 3, *p < 0.05; **p < 0.01; ***p < 0.001; ns, not significant).

To further construct a composite hydrogel system suitable for wound repair, Cu–CeO_2_ NPs were loaded into HAMA hydrogel to fabricate Cu–CeO_2_-loaded HAMA (CCH) hydrogel patches, followed by characterization of their internal structure and elemental distribution. As shown in Figure 2A, SEM images revealed that pure HAMA hydrogel exhibited a typical lamellar porous network structure with interconnected pores, which is favorable for moisture retention, nutrient exchange, and cell migration. Compared with the HAMA group, the CCH hydrogel retained a continuous porous structure, while its pore architecture appeared more open, suggesting that the incorporation of Cu–CeO_2_ NPs did not disrupt the fundamental three-dimensional network of HAMA hydrogel and may instead improve the internal pore morphology to some extent.

EDS elemental mapping further confirmed the successful loading of Cu–CeO_2_ NPs within the CCH hydrogel. As shown in Figure 2B, Ce, Cu, and O elements were detected within the hydrogel and exhibited a relatively uniform distribution, indicating that Cu–CeO_2_ NPs were well dispersed in the HAMA hydrogel matrix. These results suggest that HAMA hydrogel can serve as an effective delivery carrier for Cu–CeO_2_ NPs, providing a structural basis for their stable retention and sustained function at the wound site.

Further quantitative analysis of hydrogel pore size showed that the relative pore size was significantly increased in the CCH group compared with the HAMA group (Figure 2C). Larger pore structures are conducive to cell infiltration, nutrient diffusion, and metabolic waste removal, and also facilitate sufficient interaction between Cu–CeO_2_ NPs and the wound microenvironment (Zhang et al., 2025). Taken together, the CCH hydrogel patch exhibits a favorable porous network structure and uniform elemental distribution, providing a material structural basis for its subsequent pro-repair, anti-inflammatory, and anti-scarring effects.

### 3.3 *In vitro* biosafety evaluation of Cu–CeO_2_ NPs

**FIGURE 3:**
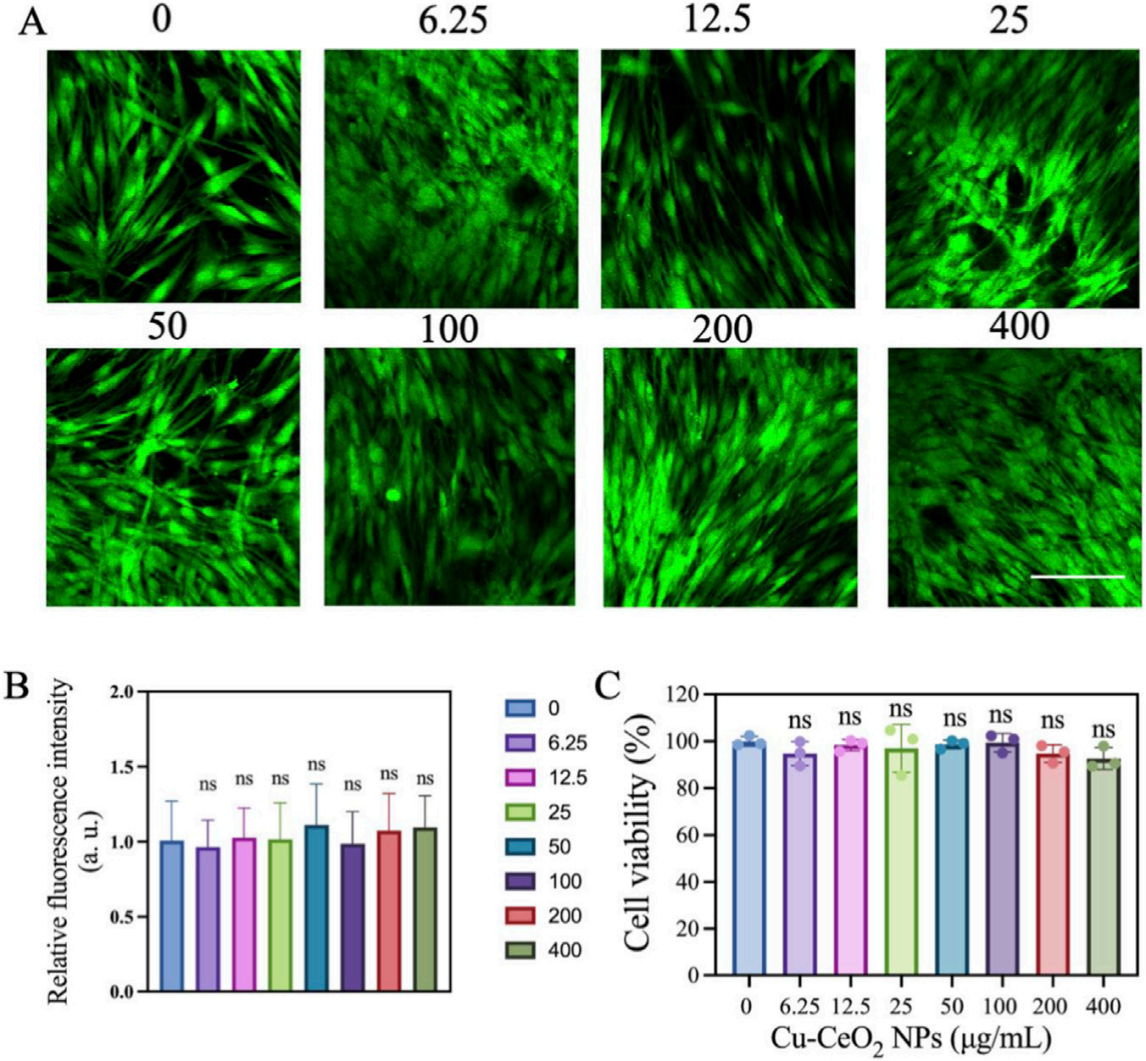
Effects of Cu–CeO_2_ nanoparticles (NPs) on fluorescence intensity and cell viability. **(A)** Live/dead staining analysis of human dermal fibroblasts (HDFs) after treatment with different concentrations (μg mL-1) of Cu–CeO_2_ NPs; scale bar = 100 μm. **(B)** Quantitative analysis of relative fluorescence intensity of cells in each group shown in **(A,C)** Cell Counting Kit-8 (CCK-8) assay for quantitative evaluation of cell viability after 48 h of treatment with different concentrations of Cu–CeO2 NPs (n = 3). All data are presented as mean ± standard deviation (SD) (n = 3, *p < 0.05; **p < 0.01; ***p < 0.001; ns, not significant).

To evaluate the biosafety of Cu–CeO_2_ NPs toward HDFs, HDFs were treated with different concentrations of Cu–CeO_2_ NPs, and their cytotoxicity was assessed by live/dead cell staining and CCK-8 assays. As shown in Figure 3A, after treatment with 0, 6.25, 12.5, 25, 50, 100, 200, and 400 μg mL-1 Cu–CeO_2_ NPs, cells in all groups exhibited obvious green fluorescence signals, relatively intact morphology, and favorable spreading, with no apparent cell shrinkage, detachment, or massive cell death observed, indicating good cytocompatibility within this concentration range.

Further quantitative analysis showed that, compared with the 0 μg mL-1 control group, the relative fluorescence intensity did not significantly decrease in any treated group (Figure 3B), suggesting that Cu–CeO_2_ NPs did not markedly affect cell viability. The CCK-8 results further confirmed that after 48 h of treatment, cell viability remained at a high level across all concentrations, with no statistically significant difference compared with the control group (Figure 3C). Even at 400 μg mL-1, no obvious reduction in viability was observed, indicating a relatively wide safety window.

Taken together, Cu–CeO_2_ NPs showed no obvious cytotoxicity within the concentration range of 0–400 μg mL-1 and exhibited favorable *in vitro* biosafety, providing a foundation for their further application in regulating fibroblast function and constructing CCH hydrogel patches.

### 3.4 Cu–CeO_2_ NPs promote HDF proliferation and migration

**FIGURE 4:**
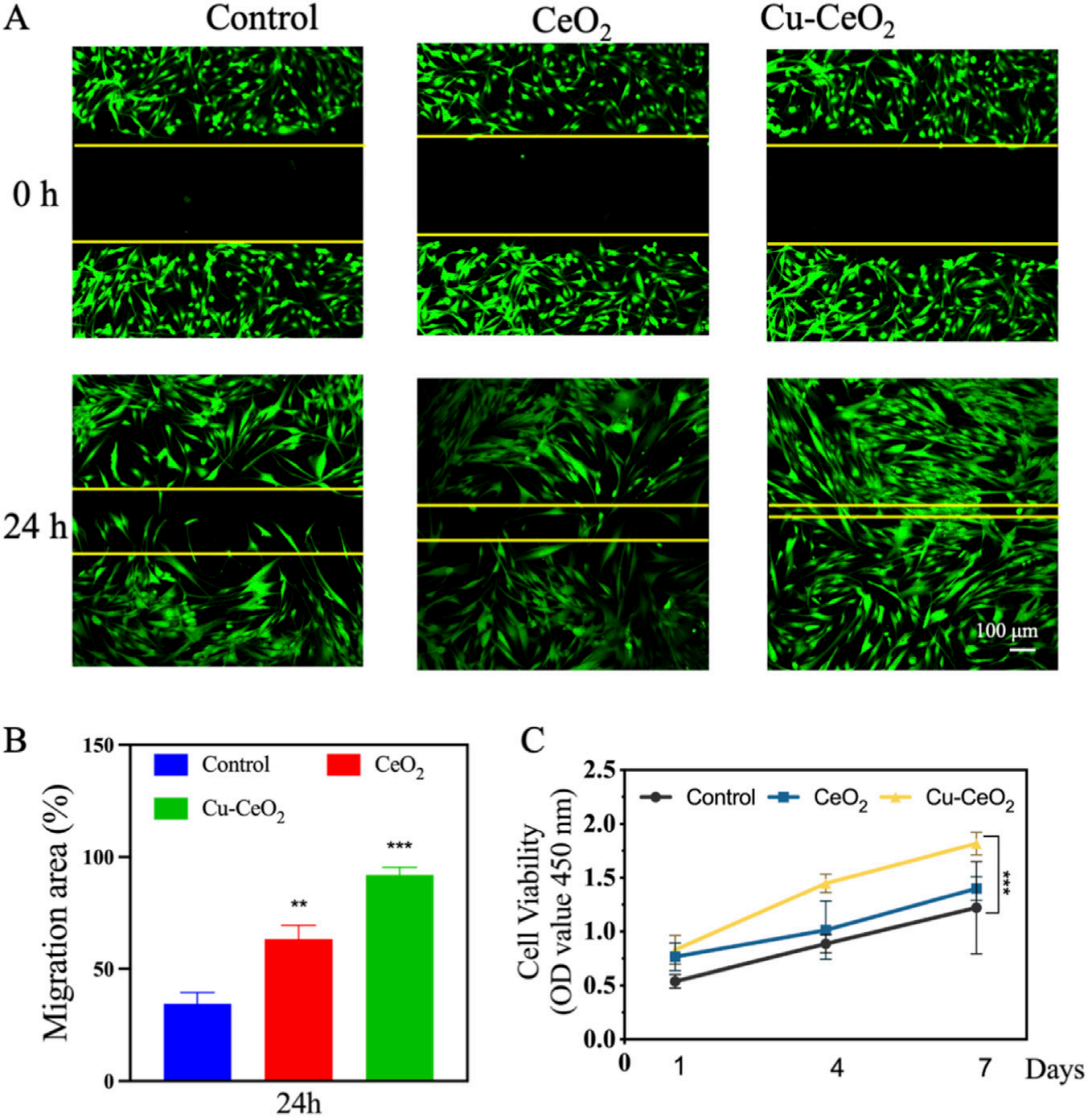
Effects of different treatments on the proliferation and migration of human dermal fibroblasts (HDFs). **(A)** Scratch assay images of the Control, cerium dioxide (CeO_2_), and Cu–CeO_2_ groups at 0 h and 24 h, showing cell migration; scale bar = 100 μm. **(B)** Quantitative analysis of migration area based on the scratch assay in **(A,C)** Cell proliferation (optical density (OD) values) of each group from day 1 to day 7. All data are presented as mean ± standard deviation (SD) (n = 3, *p < 0.05; **p < 0.01; ***p < 0.001; ns, not significant).

The migration and proliferation of fibroblasts are essential for cellular repopulation, granulation tissue formation, and matrix remodeling during wound healing (Rettinger et al., 2014). To evaluate the effects of Cu–CeO_2_ NPs on HDF functional behavior, a scratch assay was performed to assess HDF migration after treatment with different materials. As shown in Figure 4A, the scratch widths were comparable among all groups at 0 h. After 24 h of culture, the scratch area remained evident in the Control group, whereas increased cell migration into the scratched region was observed in the CeO_2_ group. Notably, the scratch area was markedly reduced in the Cu–CeO_2_ group, suggesting that Cu–CeO_2_ NPs more effectively promoted HDF migration toward the injured area.

Further quantitative analysis showed that, compared with the Control group, the migrated areas were significantly increased in both the CeO_2_ and Cu–CeO_2_ groups, with the greatest migration area observed in the Cu–CeO_2_ group (Figure 4B). CeO_2_ significantly enhanced HDF migration compared with the Control group, while Cu–CeO_2_ further strengthened this effect, indicating that Cu incorporation could further promote HDF migration based on CeO_2_. CCK-8 results showed that HDF viability gradually increased over time in all groups, with the Cu–CeO_2_ group exhibiting the highest OD value on day 7, significantly higher than those in the Control and CeO_2_ groups (Figure 4C), demonstrating that Cu–CeO_2_ NPs effectively promoted HDF proliferation.

In addition, to further assess the effects of Cu–CeO_2_ NPs on vascular-related cells, CCK-8 assays were performed to evaluate HUVEC viability after different treatments. The results showed that, compared with the Control group, the Cu–CeO_2_ group significantly increased HUVEC viability on days 1, 4, and 7 (Supplementary Figure 4), suggesting that Cu–CeO_2_ NPs may have the potential to promote endothelial cell proliferation (Salvo and Sandoval, 2022). Taken together, Cu–CeO_2_ NPs not only promoted HDF migration and proliferation but may also further improve the wound repair microenvironment by enhancing HUVEC activity, providing a cellular functional basis for subsequent wound healing promotion.

### 3.5 ROS-scavenging capacity and H_2_O_2_ decomposition-mediated oxygen generation of Cu–CeO_2_ NPs

**FIGURE 5:**
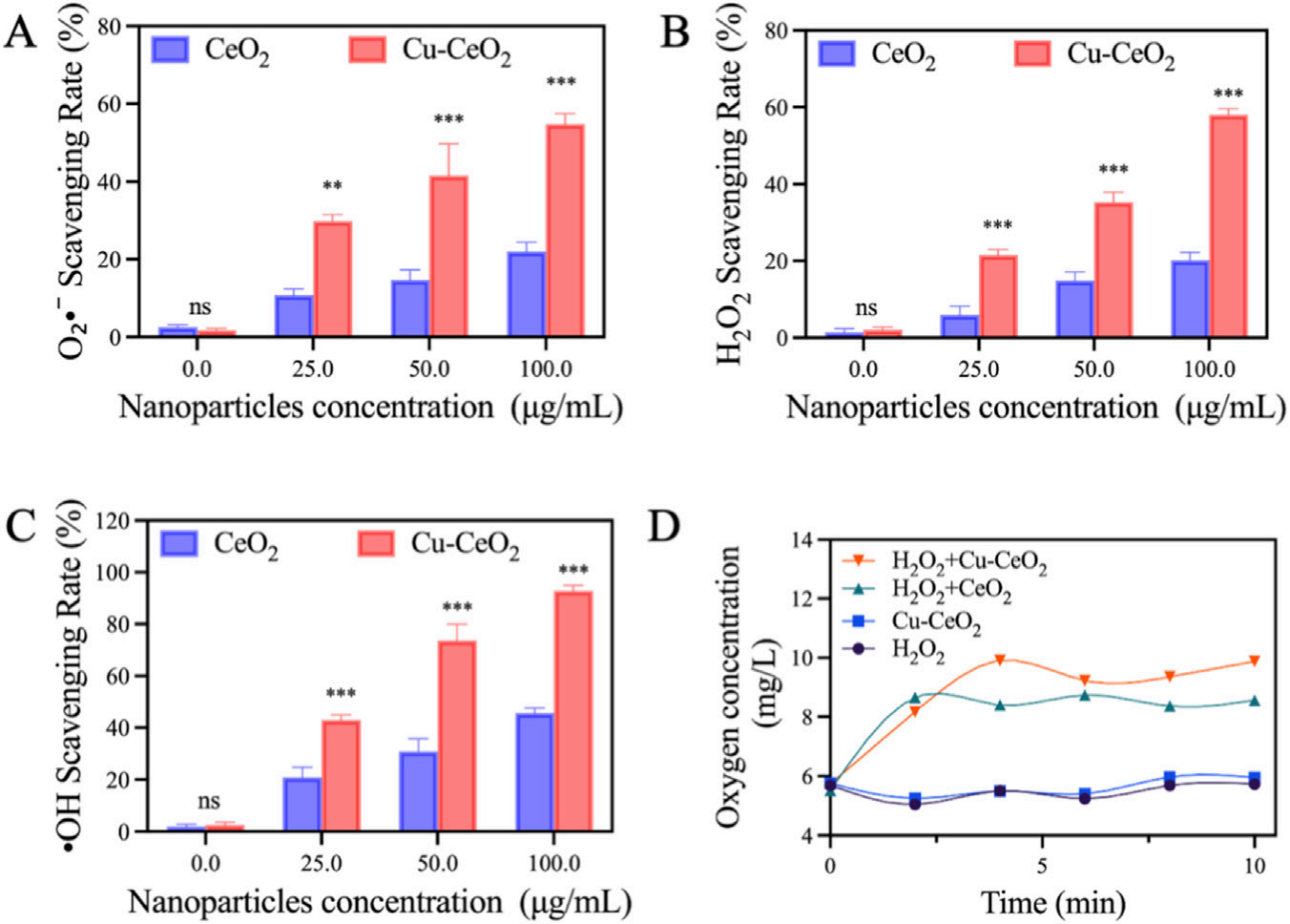
Reactive oxygen species (ROS) scavenging capacity and hydrogen peroxide (H_2_O_2_) decomposition-mediated oxygen generation of different treatments. **(A–C)** Comparison of the scavenging abilities of cerium dioxide (CeO_2_) and Cu–CeO_2_ toward **(A)** superoxide anion (O_2_•-) **(B)** hydrogen peroxide (H2O2), and **(C)** hydroxyl radical (•OH) at different concentrations (n = 3). **(D)** Comparison of oxygen concentration changes over time among different groups. All data are presented as mean ± standard deviation (SD) (n = 3, *p < 0.05; **p < 0.01; ***p < 0.001; ns, not significant).

Excessive ROS accumulation in wounds can aggravate local oxidative stress-induced damage and further sustain inflammatory responses, thereby delaying wound repair (Hunt et al., 2024; Lopes et al., 2024). To evaluate the regulatory capacity of Cu–CeO_2_ NPs in an oxidative stress microenvironment, the scavenging abilities of CeO_2_ NPs and Cu–CeO_2_ NPs against O_2_•^-^, H_2_O_2_, and •OH were examined. As shown in Figures 5A–C, both NPs exhibited certain ROS-scavenging capacity, and the scavenging rates gradually increased with increasing NP concentrations. Notably, the Cu–CeO_2_ group showed markedly higher scavenging efficiencies against O_2_•^-^, H_2_O_2_, and •OH than the CeO_2_ group at 25, 50, and 100 μg mL-1, suggesting that Cu incorporation further enhanced the antioxidant activity of CeO_2_ (Zhu et al., 2024).

Specifically, in the O_2_•^-^ scavenging assay, the scavenging rate of the Cu–CeO_2_ group exceeded 50% at 100 μg mL-1, which was significantly higher than that of the CeO_2_ group (Figure 5A). In the H_2_O_2_ scavenging assay, the Cu–CeO_2_ group also exhibited stronger H_2_O_2_-scavenging ability with an obvious concentration-dependent trend (Figure 5B). In addition, the Cu–CeO_2_ group showed potent •OH-scavenging activity, with the scavenging rate approaching 90% at 100 μg mL-1, significantly outperforming the CeO_2_ group (Figure 5C). These results indicate that Cu–CeO_2_ NPs can simultaneously scavenge multiple major ROS and possess favorable broad-spectrum antioxidant capacity (Li et al., 2024).

Further evaluation of H_2_O_2_ decomposition-mediated oxygen generation showed that oxygen concentrations changed only slightly in the H_2_O_2_ alone and Cu–CeO_2_ groups, whereas oxygen concentrations in the H_2_O_2_ + CeO_2_ and H_2_O_2_ + Cu–CeO_2_ groups increased markedly over time (Figure 5D). Among them, the H_2_O_2_ + Cu–CeO_2_ group exhibited the greatest increase in oxygen concentration, approaching 10 mg L-1 at 10 min, indicating that Cu–CeO_2_ NPs effectively catalyzed H_2_O_2_ decomposition and generated oxygen. Taken together, Cu–CeO_2_ NPs not only possess strong ROS-scavenging capacity but also promote H_2_O_2_ decomposition-mediated oxygen generation, which may help alleviate oxidative stress and local hypoxia in wounds, thereby providing a favorable microenvironment for subsequent inflammation regulation and wound repair (Han et al., 2023).

### 3.6 Cu–CeO_2_ NPs regulate macrophage polarization

**FIGURE 6:**
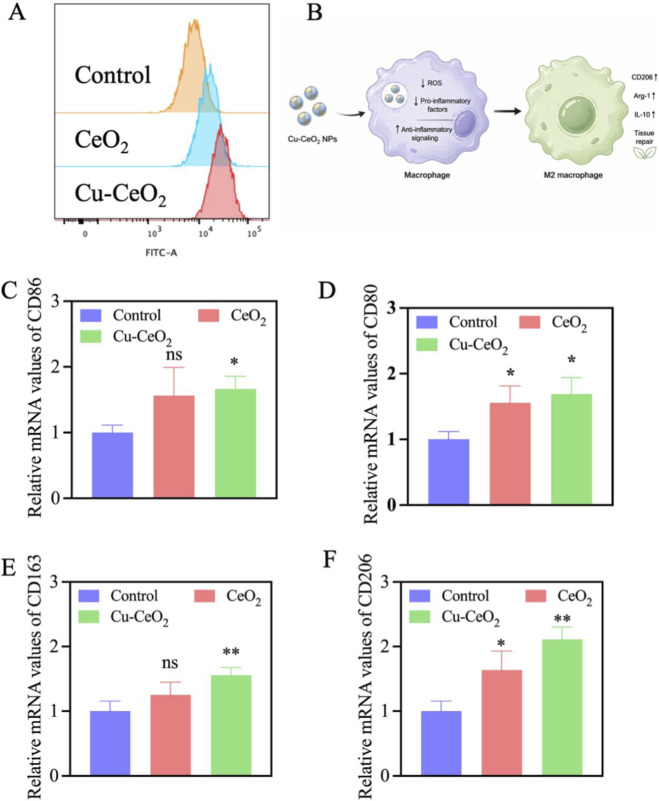
Effects of different treatments on macrophage polarization-related marker expression. **(A)** Flow cytometry analysis of the fluorescence signal distribution of M2 polarization-related markers in macrophages after treatment with Control, CeO_2_, and Cu–CeO_2_. **(B)** Schematic illustration of the mechanism by which Cu–CeO_2_ NPs promote macrophage polarization toward the M2 phenotype. Panel B was AI-assisted based on the authors’ original concept and experimental results, and was reviewed and edited by the authors. **(C–F)** Relative messenger RNA (mRNA) expression levels of CD86 **(C)**, CD80 **(D)**, CD163 **(E)**, and CD206 **(F)** in macrophages from different groups (n = 3). All data are presented as mean ± SD (n = 3, *p < 0.05; **p < 0.01; ***p < 0.001; ns, not significant).

Macrophage polarization plays a critical role in inflammation resolution and tissue repair during wound healing (Willenborg et al., 2022). M1 macrophages are generally associated with pro-inflammatory responses, whereas M2 macrophages contribute to inflammation attenuation, tissue remodeling, and wound repair (Hassanshahi et al., 2022; Zhang et al., 2026). Given the strong ROS-scavenging capacity of Cu–CeO_2_ NPs described above, we further evaluated their effects on the expression of macrophage polarization-related markers. As shown in Figure 6A, flow cytometry revealed an overall rightward shift in the fluorescence signal of M2-related markers in the Cu–CeO_2_ group compared with the Control and CeO_2_ groups, indicating enhanced M2-associated phenotypic signals after Cu–CeO_2_ NP treatment. Supplementary Figure 5 further shows the individual flow cytometry peak plots for each group.

To further explain the potential mechanism by which Cu–CeO_2_ NPs regulate macrophage polarization, a schematic illustration was generated (Figure 6B). Cu–CeO_2_ NPs may promote macrophage transition toward an M2-like phenotype by scavenging excessive ROS, reducing stimulation from the pro-inflammatory microenvironment, and enhancing anti-inflammatory and reparative signaling. Upregulation of M2-related markers, such as CD206, Arg-1, and IL-10, may contribute to inflammation resolution, tissue regeneration, and wound repair progression.

qPCR results further showed that the expression of macrophage polarization-related genes changed after different treatments. Compared with the Control group, the mRNA expression levels of CD163 and CD206 were significantly increased in the Cu–CeO_2_ group (Figures 6E,F), with CD206 showing the most pronounced elevation, suggesting that Cu–CeO_2_ NPs promoted macrophage polarization toward an M2-like reparative phenotype. Meanwhile, the expression levels of CD86 and CD80 also changed to some extent (Figures 6C,D), indicating that Cu–CeO_2_ NPs may influence the overall activation state of macrophages. Overall, Cu–CeO_2_ NPs significantly enhanced the expression of M2-related markers, particularly CD163 and CD206, providing immunomodulatory evidence for their role in alleviating inflammatory responses and promoting tissue regeneration during wound repair.

### 3.7 *In vitro* antibacterial activity of Cu–CeO_2_ NPs

**FIGURE 7:**
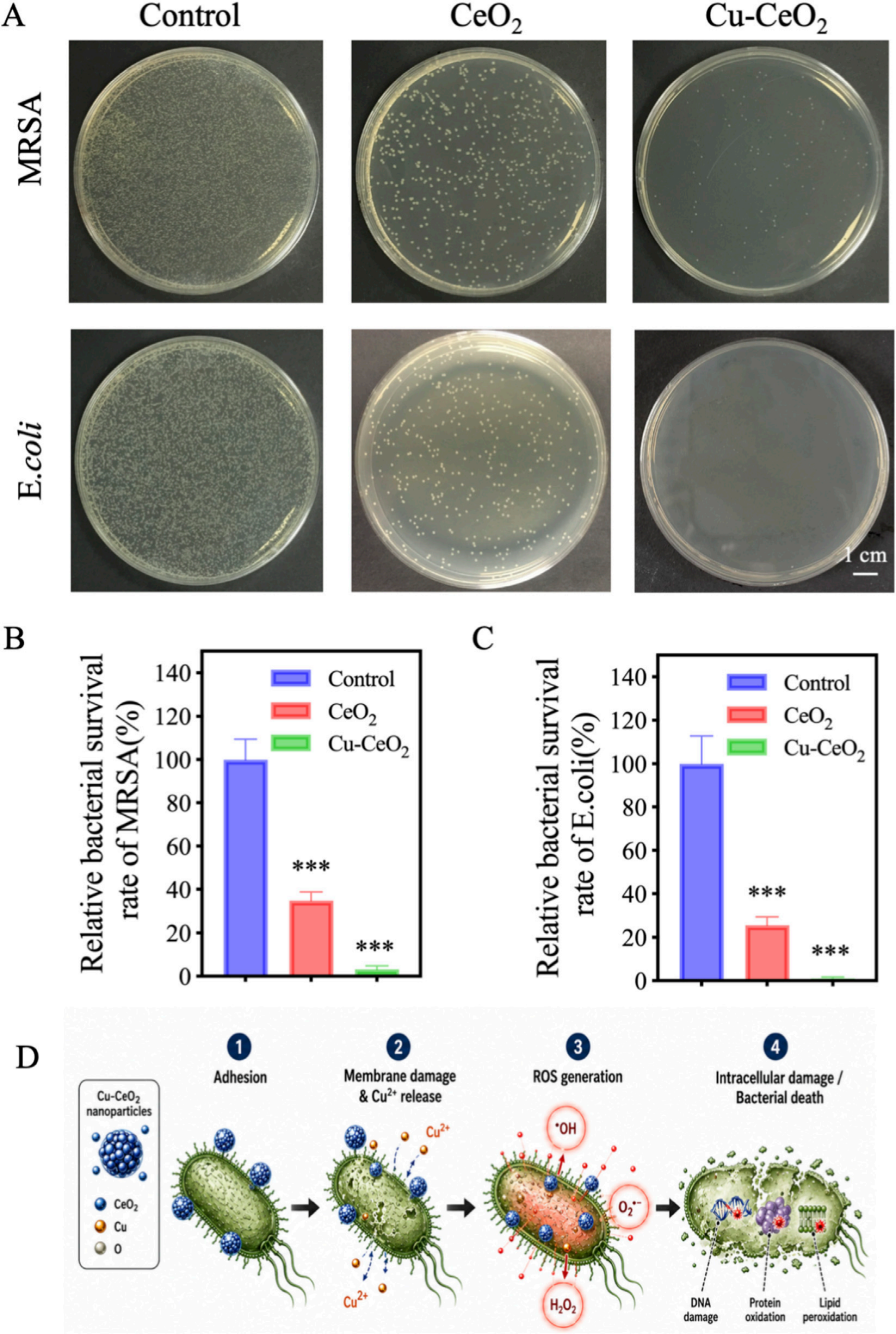
Antibacterial effects of different treatments against methicillin-resistant *Staphylococcus aureus* (MRSA) and *Escherichia coli* (*E. coli*). **(A)** Plate colony formation assay showing the surviving colonies of MRSA and *E. coli* after treatment with Control, CeO_2_, and Cu–CeO_2_; scale bar = 1 cm. **(B,C)** Quantitative analysis of relative bacterial viability of MRSA **(B)** and *E. coli* **(C)** in different treatment groups (n = 3). **(D)** Schematic illustration of the antibacterial mechanism of Cu–CeO_2_ NPs. Panel D was AI-assisted based on the authors’ original concept and experimental results, and was reviewed and edited by the authors. All data are presented as mean ± SD (n = 3, *p < 0.05; **p < 0.01; ***p < 0.001; ns, not significant).

Wound infection is one of the major factors contributing to delayed wound healing and persistent inflammation. MRSA and *E. coli* are common Gram-positive and Gram-negative bacteria in clinical wound infections, respectively (Ding et al., 2022; Vivcharenko et al., 2023; He et al., 2025). To evaluate the antibacterial activity of Cu–CeO_2_ NPs, a plate colony formation assay was performed to assess the survival of MRSA and *E. coli* after different treatments. As shown in Figure 7A, dense colonies of MRSA and *E. coli* were observed in the Control group, indicating substantial bacterial survival. The number of colonies was markedly reduced in the CeO_2_ group compared with the Control group, suggesting that CeO_2_ NPs possessed certain antibacterial activity. In contrast, the Cu–CeO_2_ group showed a further pronounced decrease in colony numbers, with only a few colonies observed on both MRSA and *E. coli* plates, indicating significant inhibitory effects against both bacteria.

Further quantitative analysis showed that, compared with the Control group, both the CeO_2_ and Cu–CeO_2_ groups significantly reduced the relative bacterial viability of MRSA and *E. coli* (Figures 7B,C). Among them, the Cu–CeO_2_ group exhibited the strongest antibacterial effect against both MRSA and *E. coli*, with relative bacterial viability approaching 0, which was significantly superior to that of the CeO_2_ group. Further analysis of the possible antibacterial mechanism suggests that the antibacterial effect of Cu–CeO_2_ nanoparticles is not simply attributable to either CeO_2_ or copper alone, but is more likely related to the combined action of both components. CeO_2_ nanoparticles themselves have redox-regulating properties, which may explain the moderate antibacterial activity observed in the CeO_2_ group in this study (Lord et al., 2021). However, compared with the CeO_2_ group, the Cu–CeO_2_ group showed a much stronger inhibitory effect against MRSA and *E. coli*, indicating that the introduction of Cu played an important role in improving the antibacterial activity. Specifically, Cu–CeO_2_ nanoparticles may first come into contact with and attach to the bacterial surface, and then disrupt membrane integrity and interfere with bacterial metabolism through Cu^2+^ release (Ermini and Voliani, 2021). At the same time, the reversible Ce^3+^/Ce^4+^ redox cycling of CeO_2_ may further take part in local ROS-related reactions and increase oxidative stress inside bacteria. Together, these effects can damage the bacterial membrane, promote protein oxidation, cause DNA damage and lipid peroxidation, and eventually lead to bacterial death. Therefore, the strong antibacterial activity of Cu–CeO_2_ nanoparticles is mainly attributed to the combined effect of Cu-mediated direct antibacterial action and the redox catalytic activity of CeO_2_. These results indicate that Cu incorporation further enhanced the broad-spectrum antibacterial capacity of CeO_2_-based NPs.

A schematic illustration was generated to summarize the possible antibacterial mechanism of Cu–CeO_2_ NPs (Figure 7D). Taken together, Cu–CeO_2_ NPs showed strong antibacterial activity against both MRSA and *E. coli*, providing an important antibacterial basis for CCH hydrogel patches in wound repair. In addition, the ability of Cu–CeO_2_ nanoparticles to decompose H_2_O_2_ and generate oxygen may also contribute to the formation of a favorable anti-infective wound microenvironment. In this study, Cu–CeO_2_ effectively catalyzed H_2_O_2_ decomposition and promoted oxygen generation, which may help relieve local hypoxia at the wound site and provide a more suitable environment for cell migration, proliferation, angiogenesis, and tissue repair. In this sense, H_2_O_2_ decomposition-driven oxygen generation and antibacterial activity are more likely to work together in this system rather than counteract each other, jointly contributing to an anti-infective, low-inflammatory, and pro-repair wound microenvironment (Mo et al., 2022).

**FIGURE 8:**
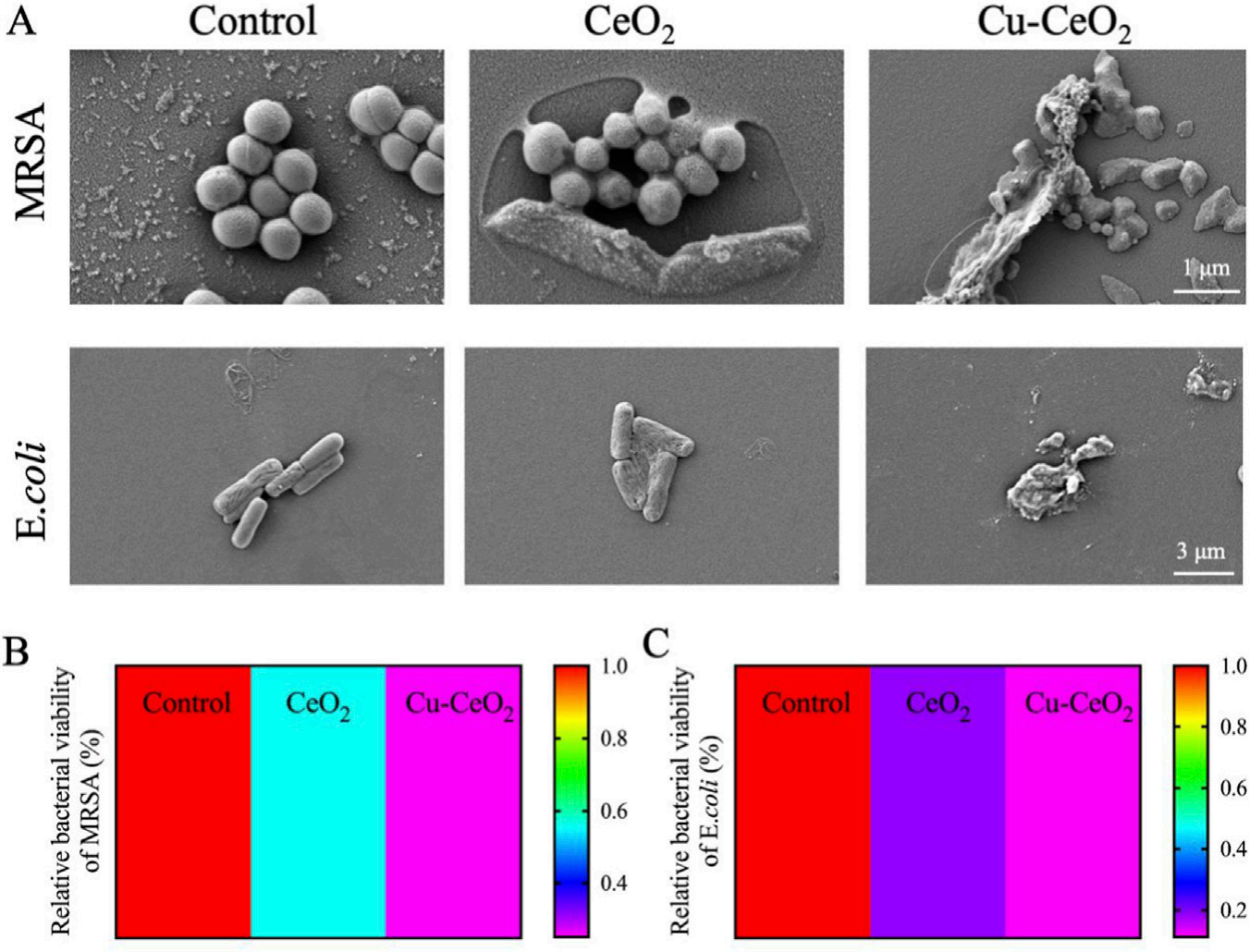
Effects of different treatments on the morphology and relative viability of MRSA and *Escherichia coli*. **(A)** SEM images showing the morphological changes of MRSA and *Escherichia coli* after treatment with Control, CeO_2_, and Cu–CeO_2_; scale bars = 1 μm for MRSA and 3 μm for *Escherichia coli*. **(B,C)** Heatmaps of the relative viability of MRSA **(B)** and *Escherichia coli* **(C)**.

To further validate the antibacterial activity of Cu–CeO_2_ NPs and observe their effects on bacterial morphology and structure, SEM was used to examine the micromorphological changes of MRSA and *E. coli* after different treatments. As shown in Figure 8A, MRSA in the Control group exhibited a typical spherical morphology with relatively intact arrangement and smooth surfaces, while *E. coli* displayed a typical rod-like structure with clear cellular contours and intact morphology. After CeO_2_ treatment, both MRSA and *E. coli* showed certain morphological alterations, including roughened bacterial surfaces and mild structural deformation, suggesting that CeO_2_ NPs caused limited bacterial damage. In contrast, bacterial morphological disruption was more pronounced in the Cu–CeO_2_ group. MRSA exhibited collapse, fragmentation, and aggregation-like changes, while *E. coli* showed loss of rod-like morphology, cellular shrinkage, and fragmentation, indicating that Cu–CeO_2_ NPs caused obvious damage to bacterial membrane structures and overall morphology.

Further heatmap visualization of relative bacterial viability showed that, compared with the Control group, the relative viability of both MRSA and *E. coli* decreased in the CeO_2_ group and was further markedly reduced in the Cu–CeO_2_ group (Figures 8B,C), consistent with the aforementioned plate colony formation results. These findings further indicate that Cu incorporation significantly enhanced the antibacterial effects of CeO_2_-based NPs. The potential mechanism may be associated with adhesion of Cu–CeO_2_ NPs to the bacterial surface, disruption of bacterial membrane integrity, Cu^2+^ release, and induction of intracellular oxidative damage, ultimately leading to bacterial structural destruction and reduced viability (Norbert et al., 2024).

In this study, Cu–CeO_2_ nanoparticles markedly inhibited the growth of MRSA and *E. coli*, while also promoting macrophage polarization toward an M2-like reparative phenotype. In contrast, M2 macrophage polarization may indirectly improve the local anti-infective wound microenvironment by promoting inflammation resolution, reducing oxidative stress, and supporting tissue repair and angiogenesis (Willenborg et al., 2022). Although M2 macrophages are not the main effector cells responsible for directly clearing bacteria, they can help reduce persistent inflammation and tissue damage, promote restoration of the wound barrier, and thereby lower the risk of continued bacterial colonization and infection spread. Thus, Cu–CeO_2_ nanoparticles may support infection control and high-quality wound repair through both direct antibacterial activity and regulation of the immune microenvironment. Taken together, Cu–CeO_2_ NPs exerted significant structural damage and antibacterial activity against both MRSA and *E. coli*, providing experimental evidence for their application in the repair of skin wounds with a high risk of infection.

### 3.8 CCH hydrogel patches promote full-thickness skin wound healing in mice

**FIGURE 9:**
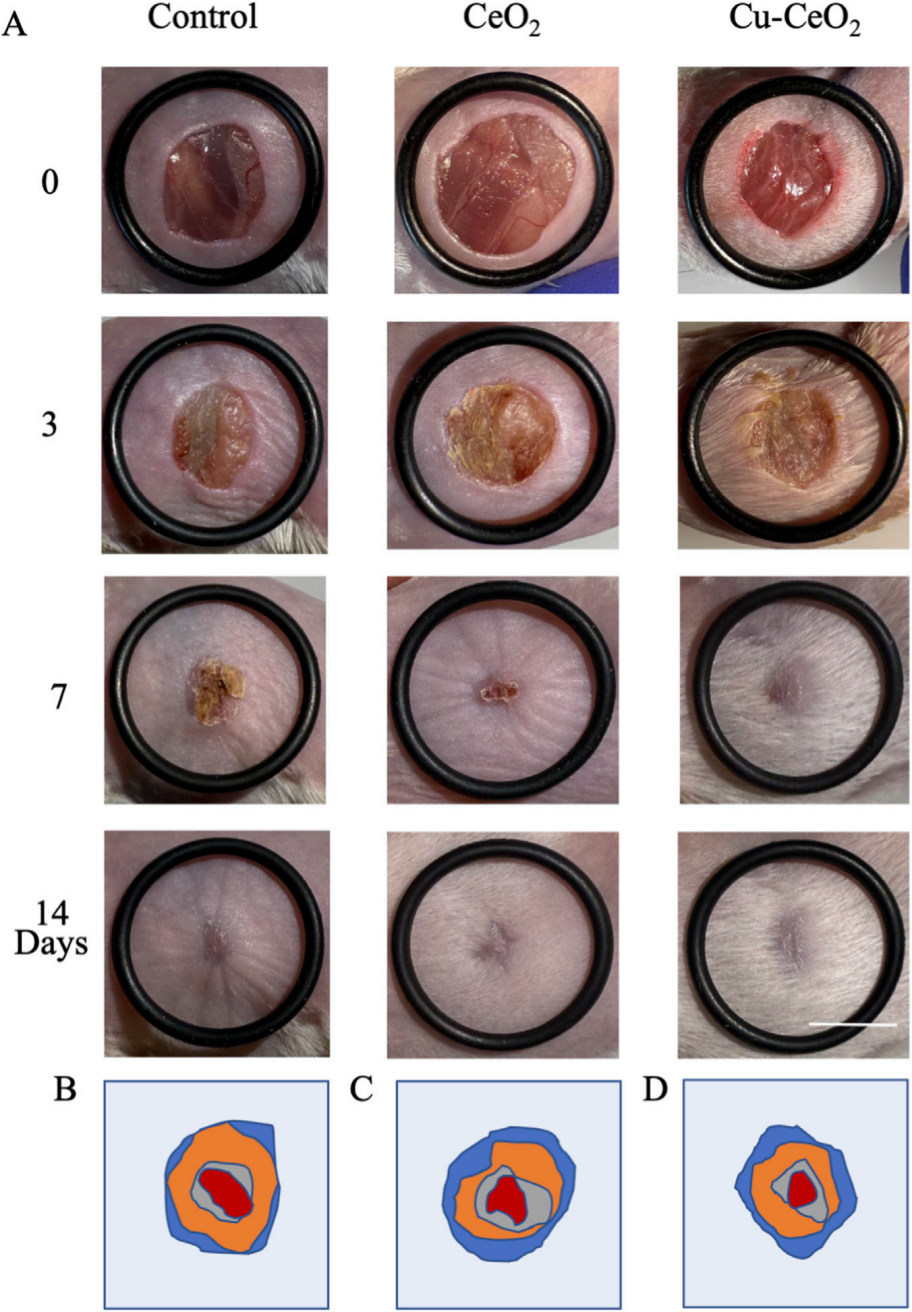
Therapeutic effects of different treatments in a mouse wound healing model. **(A)** Representative images of wound healing in different treatment groups on postoperative days 0, 3, 7, and 14; scale bar = 5 mm. **(B–D)** Heatmap analysis of wound healing in the Control **(B)**, CeO_2_ **(C)**, and Cu–CeO_2_ **(D)** groups.

To further validate the *in vivo* therapeutic efficacy of CCH hydrogel patches in wound repair, a full-thickness skin defect model was established in mice (He et al., 2025). The wounds were treated with Control, CeO_2_, or Cu–CeO_2_-related hydrogel patches, and wound healing was continuously monitored on postoperative days 0, 3, 7, and 14 (Supplementary Figures 6,7). As shown in Figure 9A, the initial wound area and status were comparable among all groups on day 0, indicating good consistency of model establishment. On day 3, all groups showed varying degrees of scab formation and wound contraction. Notably, the wound surface in the Cu–CeO_2_ group appeared drier with more uniform scab formation, without obvious exudation or signs of infection. On day 7, the Control group still showed an obvious residual wound area with scab coverage, while the wound area was further reduced in the CeO_2_ group. In contrast, wounds in the Cu–CeO_2_ group were nearly closed, and the local skin surface became relatively smooth, suggesting that Cu–CeO_2_ treatment markedly accelerated the wound repair process.

On day 14, wounds in all groups showed varying degrees of healing, but the healing quality differed among groups. Obvious scar-like traces remained in the central wound area in the Control and CeO_2_ groups, whereas the skin appearance in the Cu–CeO_2_ group was closer to that of surrounding normal tissue, with milder local scar-like changes. Further heatmap-based visualization of wound healing showed that the wound area gradually decreased over time in all groups. Among them, the Cu–CeO_2_ group exhibited the fastest wound contraction and the smallest residual wound area (Figures 9B–D; Supplementary Figure 8). To further examine the tissue structure after wound repair, H&E and Masson staining were performed on the regenerated wound tissues. H&E staining showed incomplete epidermal repair in the Control group, with a relatively disorganized tissue structure and obvious inflammatory cell infiltration. The CeO_2_ group showed some improvement compared with the Control group, with better epidermal continuity and a clearer dermal structure. In the Cu–CeO_2_ group, the epidermis was more complete, the dermal tissue was closer to normal skin, and more skin appendage-like structures could be observed. Masson staining showed uneven collagen deposition and disordered fiber arrangement in the Control group, whereas collagen fibers in the Cu–CeO_2_ group were more evenly distributed and better organized (He et al., 2025). These findings suggest that Cu–CeO_2_-loaded hydrogel patches not only promoted wound closure, but also improved the tissue structure and collagen remodeling quality after wound healing (Supplementary Figure 9). To further support the Masson staining results, quantitative and semi-quantitative analyses of collagen deposition and collagen fiber organization were performed. The results showed that collagen deposition was increased in the CeO_2_ group and was highest in the Cu–CeO_2_ group, suggesting that Cu–CeO_2_ treatment promoted extracellular matrix deposition during wound repair (Supplementary Figure 10). In addition, collagen fiber organization was semi-quantitatively evaluated based on the regularity and alignment of collagen fibers in the regenerated wound tissue. Compared with the Control group, the CeO_2_ group showed improved collagen organization, while the Cu–CeO_2_ group exhibited the most organized collagen fiber arrangement (Supplementary Figure 11). These results indicate that the CCH hydrogel patch not only promoted collagen deposition but also improved collagen fiber remodeling. Importantly, collagen deposition should be interpreted together with fiber organization, because appropriate collagen deposition is essential for restoring tissue integrity and mechanical strength, whereas excessive or disorganized collagen accumulation is closely associated with scar-like tissue formation (Jeschke et al., 2023; Mascharak et al., 2025). In this study, the Cu–CeO_2_ group showed both increased collagen content and more organized collagen fibers, suggesting that the CCH hydrogel patch may promote more orderly extracellular matrix remodeling rather than simply inducing uncontrolled collagen accumulation. This more organized collagen structure may contribute to improved healing quality and reduced scar-like changes after wound repair (Stewart et al., 2025; Hunt et al., 2024). These results indicate that CCH hydrogel patches loaded with Cu–CeO_2_ NPs significantly promoted cutaneous wound healing in mice and may help improve post-healing skin appearance and reduce scar-like changes. Combined with the aforementioned *in vitro* findings, the wound healing-promoting effect of CCH hydrogel patches may be associated with multiple synergistic functions, including promoting HDF migration and proliferation, enhancing antioxidant capacity, regulating macrophage reparative phenotypes, and inhibiting bacterial growth.

## 4 Conclusion

In this study, Cu–CeO_2_ NPs were successfully synthesized and loaded into HAMA hydrogel to construct CCH hydrogel patches with multifunctional synergistic regulatory effects. *In vitro* results demonstrated that Cu–CeO_2_ NPs exhibited favorable biocompatibility, significantly promoted HDF proliferation and migration, and enhanced HUVEC viability, providing a cellular basis for wound repair and angiogenesis. Meanwhile, Cu–CeO_2_ NPs showed strong ROS-scavenging capacity and H_2_O_2_ decomposition-mediated oxygen generation ability, which could effectively alleviate oxidative stress and local hypoxic microenvironments. In addition, they enhanced the expression of markers associated with M2-like reparative macrophage phenotypes, suggesting potential immunomodulatory and anti-inflammatory reparative effects. Antibacterial experiments further confirmed that Cu–CeO_2_ NPs significantly inhibited the proliferation of MRSA and *E. coli* and disrupted bacterial morphology and structure, thereby reducing the risk of wound infection. Furthermore, the *in vivo* full-thickness skin wound model in mice confirmed that CCH hydrogel patches accelerated wound closure, improved healing quality, and attenuated post-healing scar-like changes. Overall, CCH hydrogel patches provide a promising biomaterial strategy for high-quality repair of complex skin wounds through multiple synergistic effects, including pro-repair, antioxidative/anti-inflammatory, antibacterial, and low-scar healing effects.

## Data Availability

The original contributions presented in the study are included in the article/Supplementary Material, further inquiries can be directed to the corresponding authors.

## Glossary

HDFs: human dermal fibroblasts
HUVECs: human umbilical vein endothelial cells
HA: hyaluronic acid
HAMA: methacrylated hyaluronic acid
MRSA: methicillin-resistant *Staphylococcus aureus*
*E. coli*: *Escherichia coli*
TEM: transmission electron microscopy
HAADF: high-angle annular dark-field
HAADF-STEM: high-angle annular dark-field scanning transmission electron microscopy
STEM: scanning transmission electron microscopy
SAED: selected area electron diffraction
SEM: scanning electron microscopy
EDS: energy-dispersive X-ray spectroscopy
DLS: dynamic light scattering
ROS: reactive oxygen species
SOD: superoxide dismutase
CAT: catalase
POD: peroxidase
**H**_**2**_ **O**_**2**_: Hydrogen peroxide
**O**_**2**_ **•** ^-^: superoxide anion
•OH: hydroxyl radical
NPs: nanoparticles
**CeO**_**2**_: cerium oxide
**Fe**_**3**_ **O**_**4**_: iron oxide
**Cu** ^**2+**^: copper ion
ECM: extracellular matrix
CCK-8: Cell Counting Kit-8
OD: optical density
VEGF: vascular endothelial growth factor
CCH: Cu-CeO_2_-loaded HAMA hydrogel
PAH: poly(allylamine hydrochloride)
TEOS: tetraethyl orthosilicate
