# Supplementary material for: Cu–CeO2 NP-loaded HAMA hydrogel patches for promoting high-quality cutaneous wound healing
Source: Front Bioeng Biotechnol. 2026 Aug 5;14:1872283. doi: 10.3389/fbioe.2026.1872283 (PMC13486170; doi:10.3389/fbioe.2026.1872283)
Supplement: Supplementary file 1 [file Supplementaryfile1.docx]

**Supporting Information**

### Original Article

**Cu–CeO_2_ NP-Loaded HAMA Hydrogel Patches for Promoting High-Quality Cutaneous Wound Healing**

Jiahao Ji^1#^, Jiamin Ni^1#^, Fei Chang^1^, Like Qian^1^, Hongyi Zhang^1, 2*^, Dongli Chen^1*^

1. The Affiliated Zhangjiagang Hospital of Soochow University, Suzhou, China.

2. Department of Plastic Surgery, Ear Reconstruction Institute, Shanghai East Hospital, School of Medicine, Tongji University, Shanghai, 200120, China.

\# These authors have contributed equally to this work.

***Correspondence:**

Hongyi Zhang: drzbhy@126.com

Dongli Chen: donglichen1987@163.com

**Keywords:** nanoparticles; HAMA hydrogel; wound healing; reactive oxygen species; biomaterials

**Supplementary Figures**

**Figure S1.** SEM was used to observe the internal porous structure of the hydrogel.

**Figure S2.** Young’s modulus of HAMA and CCH hydrogels was evaluated by compression testing. Data are presented as mean ± standard deviation (n=3, *p < 0.05, **p < 0.01, ***p < 0.001, ns, not significant).

**Figure S3.** The swelling behavior and stability of HAMA and CCH hydrogels were evaluated by swelling assay.

**Figure S4.** Cell viability of HUVECs at days 1, 4, and 7 after treatment with Control, CeO₂, and Cu–CeO₂, as assessed by the CCK-8 assay (OD450 values). The results showed that, compared with the Control group, the Cu–CeO₂ group significantly promoted cell proliferation. Data are presented as mean ± standard deviation (n=3, *p < 0.05, **p < 0.01, ***p < 0.001).

**Figure S5.** Flow cytometry analysis of the fluorescence signal distribution of M2 polarization-related markers in macrophages after treatment with Control, CeO₂, and Cu–CeO₂.

**Figure S6.** (A) Structure of the hydrogel patch. (B) Established mouse wound model. (C) Application of the hydrogel patch to the mouse wound.

**Figure S7.** Schematic illustration of the wound healing process in mice. Figure S7 was AI-assisted based on the authors’ original concept and experimental results, and was reviewed and edited by the authors.

**Figure S8.** Effects of different treatments on wound healing area in mice. The relative wound area (normalized to 100% at day 0) was compared among the Control, CeO₂, and Cu–CeO₂ groups at postoperative days 0, 3, 7, and 14. The results showed that, compared with the Control group, the Cu–CeO₂ group exhibited a faster wound healing trend at all time points. All data are presented as mean ± standard deviation (SD) (n=3, *p < 0.05; **p < 0.01; ***p < 0.001; ns, not significant).

**Figure S9.** H&E and Masson staining were used to evaluate wound tissue regeneration in different treatment groups. Scale bar = 150 μm.

**Figure S10.** Analysis of collagen deposition in wound tissues. The Cu-CeO₂ group showed the highest collagen deposition. All data are presented as mean ± standard deviation (SD) (n=3, *p < 0.05; **p < 0.01; ***p < 0.001; ns, not significant).

**Figure S11.** Analysis of collagen fiber organization in wound tissues. The Cu-CeO₂ group showed more organized collagen fibers. All data are presented as mean ± standard deviation (SD) (n=3, *p < 0.05; **p < 0.01; ***p < 0.001; ns, not significant).
